# Thiourea-catalyzed Diels–Alder reaction of a naphthoquinone monoketal dienophile

**DOI:** 10.3762/bjoc.9.158

**Published:** 2013-07-12

**Authors:** Carsten S Kramer, Stefan Bräse

**Affiliations:** 1 Karlsruhe Institute of Technology (KIT), Institute of Organic Chemistry, Fritz-Haber-Weg 6, D-76131 Karlsruhe; Tel: (+49) 721608-48581; 2 Karlsruhe Institute of Technology (KIT), Institute of Toxicology and Genetics, Hermann-von-Helmholtz-Platz 1, D-76344 Eggenstein-Leopoldshafen, Germany

**Keywords:** beticolin 0, Diels–Alder reaction, natural product, organocatalysis, thiourea catalysis, total synthesis

## Abstract

A variety of organocatalysts were screened for the catalysis of the naphthoquinone monoketal Diels–Alder reaction. In this study we found that Schreiner's thiourea catalyst **10** and Jacobson's thiourea catalyst **12** facilitate the cycloaddition of the sterically hindered naphthoquinone monoketal dienophile **3** with diene **4**. The use of thiourea catalysis allowed for the first time the highly selective synthesis of the exo-product **2a** in up to 63% yield. In this reaction a new quaternary center was built. The so formed cycloaddition product **2a** represents the ABC tricycle of beticolin 0 (**1**) and is also a valuable model substrate for the total synthesis of related natural products.

## Introduction

In the past few years, many different types of organocatalysts were found to accelerate a variety of reactions. Due to the value of the Diels–Alder reaction for the synthetic community, different organocatalysts have been developed to catalyze this atom-economical cycloaddition in a highly enantioselective fashion. Thereby, chiral amines, heterocyclic carbenes, guanidines, thioureas, amidinium ions, diols, and Brønsted acids showed their value to give densely functionalized Diels–Alder products in high selectivities [1–7]. In addition, some organocatalysts enabled even the formation of quaternary centers in Diels–Alder cycloadditions [3,8].

**Scheme 1:**
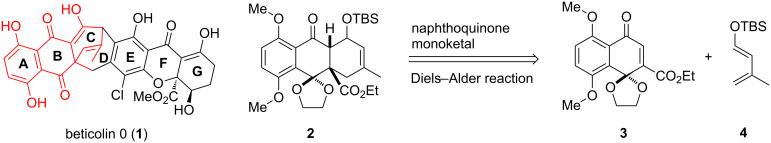
Retrosynthetic dissection of the ABC-ring system of beticolin 0 (**1**).

In our recent studies towards a synthetic access to the natural product beticolin 0 (**1**) [9] (Scheme 1), we have found that naphthoquinone monoketals are suitable building blocks for our designed synthesis [9]. In our retrosynthetic approach, the naphthoquinone monoketal dienophile **3** was found to undergo cycloaddition with diene **4** to tricycle **2**, which represents the ABC-ring system of beticolin 0 (**1**) and other beticolins [10].

We found that dienophile **3** and diene **4** can undergo a cycloaddition by applying either thermal conditions or microwave irradiation to these both compounds (Table 1) [9]. Thereby, a mixture of regioisomers was always isolated. Regioisomer **5** was obtained diastereoselectively pure, the other isomer was received in a 3:1 (Table 1, entry 1) or 4:1 (Table 1, entry 2) mixture of **2a** and **2b**.

**Table 1:** Preliminary studies towards the ABC tricycle of beticolin 0 (**1**) [9].

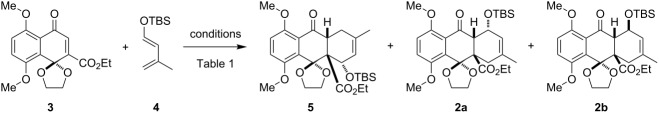	

Entry	Product/Yield/d.r.

1^a^	**2a/b** (69%, d.r. 3:1), **5** (16%)
2^b^	**2a/b** (81%, d.r. 4:1), **5** (16%)

^a^Conditions: µW, 300 W, 0.5 h, 170 °C. ^b^Conditions: 170 °C, 1.5 h.

The rather harsh conditions and the lack of selectivity in the cycloaddition of dienophile **3** and diene **4** motivated us to find an appropriate catalyst system. Since the 1,3-dioxolane ring of **3** is prone to Lewis acid mediated ketal opening [11], we had to exclude metal catalysts and thus screened a variety of different organocatalysts.

## Results

For the screening of an appropriate organocatalyst, dienophile **3** and diene **4** were stirred together at room temperature with an equimolar amount of the prospective organocatalyst in the absence of any solvent. The catalytic performance was classified by the acceleration of the reaction time compared to the uncatalyzed reaction (Table 2, entry 1).

**Table 2:** Catalyst screening for the naphthoquinone monoketal Diels–Alder reaction.

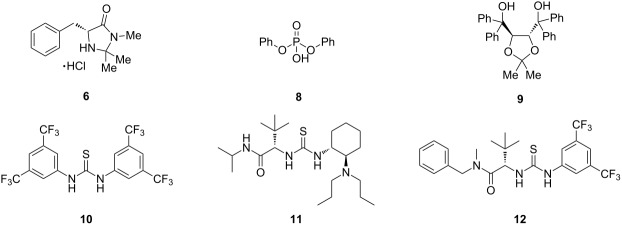		

Entry	Catalyst^a^	Catalyst performance^b^

1	–	0
2	**6**	0
3	L-proline	+
4	**8**	0
5	**9**	++
6	**10**	+++
7	**11**	++
8	**12**	+++

^a^Dienophile **3** was stirred at rt with diene **4**. In the case of entries 2–8, one equiv catalyst was added. ^b^Performance assessment: 0 = no acceleration, trace amounts of product appear after several weeks; + = slight acceleration, no full conversion even after 2 months; ++ = full conversion between 1–2 months; +++ = full conversion in less than one month.

At first, the use of MacMillan's imidazolidinone organocatalyst **6** [12] was examined, but no catalytic effect was observed (Table 2, entry 2). The usage of L-proline as a bifunctional catalyst only gave a slight improvement compared to the uncatalyzed reaction (Table 2, entry 3). Whereas the addition of one equivalent of Brønsted acid **8** had no effect (Table 2, entry 4), the addition of TADDOL (**9**) improved the reaction speed (Table 2, entry 5). By the use of Schreiner's catalyst **10** [7], the highest conversion speed was observed (Table 2, entry 6). After this finding, Jacobsen's thioureas **11** [13] (Table 2, entry 7) and **12** [14] (Table 2, entry 8) were also tested whereby the 3,5-bis(trifluoromethyl)phenyl-substituted thiourea **12** was superior to catalyst **11**. The fact that thioureas as well as diol **9** improved the reaction speed was coherent, since both catalysts are well known to catalyze the Diels–Alder reaction by hydrogen-bond catalysis [1].

Subsequently, we examined the Diels–Alder reaction of dienophile **3** with diene **4** with 10 mol % of the superior catalysts **10** and **12** (Table 3). In this way, the desired tricycle **2a** was obtained by usage of Schreiner's catalyst **10** in 63% yield (Table 3, entry 1). To our delight and in contrast to the uncatalyzed reaction conditions (Table 1), just one regioisomer was isolated. In addition, the cycloaddition with thiourea catalyst **10** afforded exclusively the exo-Diels–Alder product **2a**, whereby thermal conditions afforded an inseparable mixture of **2a** and **2b** besides the separable product **5**. The usage of Jacobsen's catalyst **12** also gave the neat exo-Diels–Alder product **2a** without any accompanying isomers, but with lower yield (29%) and no remarkable ee value (6% ee, determined by HPLC) (Table 3, entry 2). The reaction of an electronically deactivated and sterically demanding dienophile, in which the ethyl ester was replaced with a CH_2_OTBS group, failed on using catalyst **10** and diene **4**.

**Table 3:** Thiourea-catalyzed naphthoquinone monoketal Diels–Alder reaction.

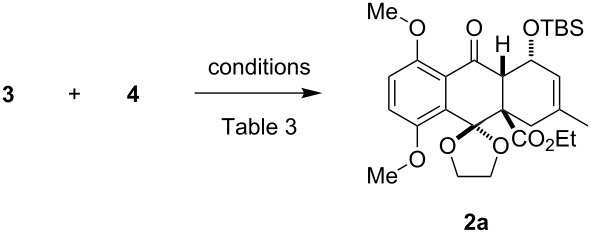		

Entry	Catalyst	Yield

1^a^	**10**	63% (29% recov. SM)
2^a^	**12**	29% (53% recov. SM) 6% ee

^a^Dienophile **3** was stirred together with diene **4** and 10 mol % catalyst at rt for 28 d.

## Discussion

**Figure 1:**
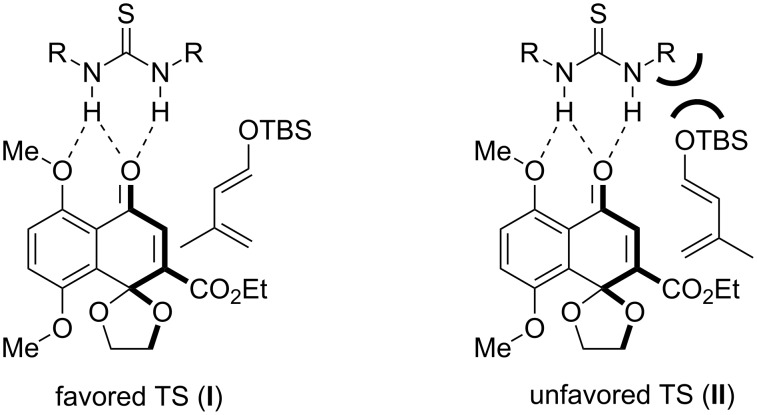
Proposed favored and disfavored transition states during the thiourea catalyzed Diels–Alder reaction of dienophile **2** and diene **3**. R = 3,5-bis(trifluoromethyl)phenyl.

It is well known that thiourea **10** facilitates the Diels–Alder reaction by activation of the dienophile with hydrogen-bond catalysis [7]. The ester group in **3** is apparently the most basic carbonyl group in the molecule and the ester group should undergo hydrogen-bonding with **10**. However, the resulting product **5** was not observed upon use of **10** or **12**. To explain the observed regioselectivity, we can assume that the thiourea catalyst is more attracted to the carbonyl function within the monoketal naphthoquinone system due to steric reasons. Whereas the 1,3-dioxalane ring hampers bonding of the catalyst on the ester group, the aromatic methoxy group could facilitate catalyst attraction by supporting hydrogen bonds [6,15] (Figure 1). In addition, the 3,5-bis(trifluoromethyl)phenyl group could be involved in the substrate bonding [16]. That could explain the lower reaction acceleration of catalyst **11** compared to the 3,5-bis(trifluoromethyl)phenyl-substituted thiourea catalysts **10** and **12**. Since the bulky OTBS-group from **4** points out of the favored transition state **I**, the high exo-selectivity for the Diels–Alder reaction can be explained. In the disfavored transition state **II** the bulky OTBS-group conflicts with the catalyst–substrate complex.

Many organocatalytic reactions are known for their long reaction times because of the relatively weak interactions between catalyst and substrate. Also the naphthoquinone monoketal Diels–Alder reactions were quenched after 28 d. After that time no further conversion was observed and some starting material could be recovered. Nevertheless it has to be considered that the catalyst facilitates the construction of a very congested quarternary center in alpha-position to the obstructed spiroketal center, rendering the long reaction times valid.

## Conclusion

In summary, we screened a variety of different organocatalysts to promote the naphthoquinone monoketal Diels–Alder reaction. Thereby we found that thioureas **10** and **12** facilitate the cycloaddition of the sterically hindered dienophile **3** with diene **4** in up to 63% yield in high selectivity. Although long reaction times were a drawback, this represents the first time and the sole method for the generation of neat exo-product **5a**, bearing a newly formed quaternary center. Additionally, tricycle **5a** is seen as a valuable intermediate for the total synthesis of beticolin 0 (**1**) and related natural products.

## Experimental

IR: spectra were measured with a Bruker IFS 88 spectrometer, and wave numbers are given in cm^−1^. MS (EI): mass spectra were measured with a Finnigan MAT 95. NMR: Spectra were recorded on a Bruker Avance 400 or Avance DRX 500 spectrometer in the solvents indicated; ^1^H and ^13^C chemical shifts (δ) are given in parts per million relative to TMS, coupling constants (*J*) in hertz. The solvent signals were used as references and the chemical shifts were converted to the TMS scale. Unless stated otherwise, all commercially available compounds (Acros, Fluka, Aldrich) were used as received.

**Ethyl 4-((*tert*-butyldimethylsilyl)oxy)-5,8-dimethoxy-2-methyl-10-oxo-4,4a,9a,10-tetrahydro-1*H*-spiro[anthracene-9,2'-[1,3]dioxolane]-9a-carboxylate (2a)**: In a small flask dienophile **3** (23.1 mg, 69.2 µmol) was covered with diene **4** (250 μL). The mixture was stirred together with Schreiner's catalyst **10** (3.50 mg, 6.92 µmol) at room temperature for 28 d. Purification by column chromatography (silica gel, ethyl acetate/cyclohexane 2:8 to 3:7) afforded product **2a** (23.3 mg, 43.7 µmol, 63% yield) as a colorless oil as well as starting material **3** (6.73 mg, 20.1 µmol, 29% yield). If necessary, also diene **4** could be recovered by Kugelrohr distillation before column chromatography was done. *R*_f_ 0.48 (ethyl acetate/cyclohexane 1:1); ^1^H NMR (400 MHz, CDCl_3_) δ 6.99 (d, *J* = 9.1 Hz, 1H), 6.87 (d, *J* = 9.1 Hz, 1H), 5.44 (s, 1H), 4.60 (s, 1H), 4.27–4.08 (m, 5H), 3.98–3.88 (m, 1H), 3.77 (s, 3H), 3.73 (s, 3H), 3.68 (d, *J* = 5.1 Hz, 1H), 2.76 (d, *J* = 17.5 Hz, 1H), 2.14 (d, *J* = 17.8 Hz, 1H), 1.58 (s, 3H), 1.24 (t, *J* = 7.1 Hz, 3H), 0.78 (s, 9H), 0.02 (s, 3H), 0.00 (s, 3H); ^13^C NMR (101 MHz, CDCl_3_) δ 193.5, 172.0, 151.7, 151.3, 133.4, 129.9, 126.0, 125.3, 117.7, 115.0, 109.8, 67.9, 67.6, 67.2, 61.7, 58.3, 57.3, 57.1, 52.8, 32.6, 26.0, 25.8, 23.4, 18.3, 14.3, −4.3, −4.61; IR (thin film) ν_max_: 1710, 1477, 1269, 1208, 1002, 959, 920, 836, 805, 776, 723 cm^−1^; EIMS *m*/*z*: 532.2, 487.2, 476.2, 475.2, 413.1, 403.1, 402.1 358.1, 357.1; HRMS–EI (*m*/*z*): [M]^+^ calcd for C_28_H_40_O_8_Si, 532.2491; found, 532.2493.
